# Controlling Blown Pack Spoilage Using Anti-Microbial Packaging

**DOI:** 10.3390/foods6080067

**Published:** 2017-08-12

**Authors:** Rachael Reid, Andrey A. Tyuftin, Joe P. Kerry, Séamus Fanning, Paul Whyte, Declan Bolton

**Affiliations:** 1 Food Safety Department, Teagasc Food Research Centre, Ashtown, Dublin 15, Ireland; rachel.reid@teagasc.ie; 2 Food Packaging Group, School of Food & Nutritional Sciences, University College Cork, College Road, Cork, Ireland; a.tiuftin@ucc.ie; 3 School of Public Health, University College Dublin, Belfield, Dublin 4, Ireland; joe.kerry@ucc.ie (J.P.K.); sfanning@ucd.ie (S.F.); paul.whyte@ucd.ie (P.W.)

**Keywords:** blown pack spoilage, *C. estertheticum*, antimicrobials, gelatine films, edible coatings, active food packaging

## Abstract

Active (anti-microbial) packaging was prepared using three different formulations; Auranta FV; Inbac-MDA and sodium octanoate at two concentrations (2.5 and 3.5 times their minimum inhibitory concentration (MIC, the lowest concentration that will inhibit the visible growth of the organisms) against *Clostridium estertheticum*, DSMZ 8809). Inoculated beef samples were packaged using the active packaging and monitored for 100 days storage at 2 °C for blown pack spoilage. The time to the onset of blown pack spoilage was significantly (*p* < 0.01) increased using Auranta FV and sodium octanoate (caprylic acid sodium salt) at both concentrations. Moreover, sodium octanoate packs had significantly (*p* < 0.01) delayed blown pack spoilage as compared to Auranta FV. It was therefore concluded that Auranta FV or sodium octanoate, incorporated into the packaging materials used for vacuum packaged beef, would inhibit blown pack spoilage and in the case of the latter, well beyond the 42 days storage period currently required for beef primals.

## 1. Introduction

Blown pack spoilage (BPS), characterised by a putrid smell (H_2_S) with a metallic sheen on the meat with or without gas production, occurs in correctly chilled batches (0 to 2 °C) after four to six weeks and is caused by psychrophilic *Clostridium* spp. [1]. Although *Clostridium algidicarnis*, *Clostridium frigoris*, *Clostridium bowmanii*, *Clostridium frigidicarmis* and *Clostridium ruminantium* have been associated with meat spoilage, they do not produce gas [2,3,4]. Blown pack spoilage is usually caused by other *Clostridium* spp., including *C. estertheticum* and *C. gasigenes*, which produce large volumes of gas, primarily carbon dioxide [3,5,6,7].

A low percentage of beef primals are consistently contaminated with *C. estertheticum* or *C. gasigenes* spores [1] and previous research by Moschonas et al. [6] showed that low contamination levels (as low as 1 spore per cm^2^) are sufficient to cause spoilage. When beef is vacuum packaged, the shrinkage step (e.g., 90 °C for 3 s) activates the spores [8] which germinate and grow in the anaerobic and low temperature environment in which beef primals and sub-primals are typically stored. Spoilage may occur as soon as two weeks but typically after four to six weeks.

As there are no specific interventions available to prevent BPS, control is currently reliant on reducing contamination using sporicidal agents, such as peroxyacetic acid, to disinfect the plant and equipment. Moreover, apart from lactic acid treatment of carcasses, processors are not legally permitted to apply chemical treatments to beef products. Active packaging provides a vehicle by which anti-microbials can be applied to inhibit bacterial growth on beef. Previous research has demonstrated a reduction in *Lactobacillus helveticus* and *Brochothrix thermosphacta* counts on vacuum-packed beef using a polyethylene-based plastic film incorporating nisin [9]. Oregano and garlic have also been incorporated into whey-protein based films to control *Salmonella enteritidis*, *Listeria monocytogenes*, *Escherichia coli*, *Staphylococcus aureus* and *Lactobacillus plantarum* [10].

Antimicrobial compounds in active packaging films may be incorporated into the film or coated in a carrier matrix onto the inner surface of the film [11]. Carrier matrices include edible polymers such as gelatine. Commercially available antimicrobials Auranta FV (AFV) (composed of bioflavonoids, citric, malic, lactic, and caprylic acids), Inbac-MDA (IMDA) (composed of sodium diacetate, malic acid, mono and diglycerides of fatty acids, salt and excipients) and sodium octanoate (SO) are considered to be safe, with potential application in active packaging systems [12]. Moreover, they are odourless and do not adversely affect other sensory attributes of food, such as taste or texture. Their application in a gelatine carrier to inhibit anaerobic bacteria has been previously demonstrated [13]. The objective of this study was to test three different formulations; AFV, IMDA and SO at two concentrations, 2.5% and 3.5% times their MIC against *C. estertheticum*, as agents in active packaging to prevent the growth of this bacteria.

## 2. Materials and Methods

### 2.1. Materials

The antimicrobials used in this study included AFVand SO which were obtained fromSigma-Aldrich, Gillingham, Dorset, UK and IMDA which was purchased from Envirotech Innovative Products Ltd, Ardee, County Louth, Ireland. Glycerol (KB Scientific Ltd, Cork, Ireland) was used as a plasticizer and beef gelatine 100 bloom (Healan ingredients, York, UK) was used as the matrix material for all film forming solutions. Beef sub-primal striploins were purchased from a local beef supplier. Conventional vacuum heat shrinking pouches (265 × 290 mm, 50 µm; water vapour transmission rate of 50 g/m^2^ day) were supplied by Cryovac, Trade Name BB3055X (Sealed Air W.R. Grace Europe Inc., Lausanne, Switzerland) and used as industry standard materials for coatings and meat packaging trials.

### 2.2. Plasma Treatment

In order to increase the hydrophilicity of the Low-density polyethylene (LDPE) inner part of the vacuum pouches; cold plasma treatment was carried out using a Dielectric-Barrier Discharge plasma system prior to the application of the antimicrobial coatings. Briefly, pouches were cut to a size of 190 × 500 mm and the surface of the LDPE side of the laminate pouches were plasma treated at atmospheric pressure using atmospheric air. The plasma source consisted of two circular aluminium plate electrodes (outer diameter = 158 mm). The top dielectric barrier was a perspex dielectric barrier (10 mm thickness) and the bottom dielectric barrier was a polypropylene sheet (5 mm thickness). When the potential across the gap reached the breakdown voltage, the dielectric barrier prevented the arc transition and homogenised the micro-discharges. The voltage applied was 75 ± 0.2 kV which was obtained from a step-up transformer (Phoenix Technologies, Inc., Campbell, CA, USA) using a variac. The input of 230 V, 50 Hz was given to the primary winding of high voltage step-up transformer from the mains supply. The samples were plasma treated for 60 s in three different places to cover the entire film area, leaving only approximately 5 cm from the edge of the film (high voltage electrode was placed 1 cm above the film).

Plasma treatment was carried out on the film samples. Following treatment, plasma treated samples were placed in Ziploc^®^ plastic bags to protect the films from antistatic and dust particles. The water droplet test was used to determine the activation of the surface. Plasma treated film samples were then coated with water-based gelatine coatings containing the test antimicrobials.

### 2.3. Coatings Preparations and Packaging of Beef

#### 2.3.1. Preparation of Film Forming Solutions and Coatings

Exactly 25 g of dry beef gelatine was dissolved in 475 mL of distilled water (5% w/w) in a 500 mL flask by heating at 90 °C in a shaking water bath (SW23, Julabo USA INC., Allentown, PA, USA) for 30 min during which 8.25 g (33% w/w) of glycerol was added under constant stirring. This solution was cooled to 40 °C in a waterbath, before the addition of the antimicrobials. The antimicrobial solutions were prepared as follows; 25.41 mL and 35.55 mL of AFV (liquid), 19.05 g and 26.65 g of IMDA (solids) and 19.0 g and 26.65 g of SO (solids) dissolved in 50 mL of distilled water before addition to the gelatine solutions to give final concentrations for each treatment of 2.5 and 3.5 times the MIC against *C. estertheticum*. These concentrations were selected to ensure the antimicrobials were present at concentrations at which they were effective against *C. estertheticum*, allowing for a dilution effect, etc. when working in food systems. Each solution was then cast on conventional polyamide/Low-density polyethylene (PA/LDPE) films using a Micron II film applicator (Gardco, Pompano Beach, FL, USA), sealed and dried at 20 °C for 48 h. The thickness of each resultant gelatine coated film was measured using a digital micrometer—Käfer Digital Thickness gauge (Käfer Messuhrenfabrik GmbH & Co., Villingen-Schwenningen, Germany) and ranged from 5 to 25 µm.

#### 2.3.2. Vacuum Packaging of Beef

Conventional PA/LDPE films (BB3055X, Cryovac, Sealed Air Ltd, St Neots, UK) coated with the active gelatine-based antimicrobials were detached from the flat surface on which they were coated, the edges of each laminate sample were cleaned with water and/or ethanol and dried. Each film was then heat-sealed to form a pouch (approx. 170 × 220 mm) using a Webomatic type D463 (Webomatic Vacuum Packaging Systems, Bochum, Germany) with the sealing time set at 2.7 s. In order to avoid adhesion between the coated films, sterile food grade aluminium foil was placed between the films prior to sealing. Exactly 15 samples (5 in triplicate) were prepared for each antimicrobial-concentration combination. Untreated PA/LDPE film was used for the control pouches.

### 2.4. Inoculation of Beef Samples and Monitoring for Blown Pack Spoilage

#### 2.4.1. Preparation of Blown Pack Spoilage *C. estertheticum*

Reference strain *C.estertheticum* subsp. *estertheticum* (DSMZ 8809^T^), was purchased as a freeze dried culture from the Deutsche Sammlung von Mikroorganismen und Zellkulturen GmbH (DSMZ, Braunchweig, Germany). The strain was revived under anaerobic conditions in 10 mL pre-reduced Peptone Yeast Extract Glucose Starch (PYGS) broth [14] and incubated for 3 weeks at 4 °C.

#### 2.4.2. Preparation of Spore Inocula

Spore concentrates were prepared by transferring 5 mL of exponentially growing culture to 100 mL of pre-reduced peptone yeast extract glucose starch (PYGS) broth [14] and incubating at 4 °C for a minimum of 3 months to promote sporulation. Prior to inoculation all media were pre-reduced in an anaerobic cabinet for 24 h (Don Whitley Scientific Ltd, Shipley, UK) under an atmosphere of 100% carbon dioxide at 20 °C. Spores were harvested using the method described by Moschonas et al. [6]. Briefly, spore suspensions were recovered by centrifugation (7500 g, 4 °C, 10 min) and washed with saline (0.85% NaCl in sterile water). This was repeated 3 times. The washed spore suspension was then sonicated (40 kHz, 15 min) in an ultrasonic waterbath (VWR International, Dublin, Ireland) and centrifuged/washed as described above (three sonification/centrifugation/wash cycles) before final suspension in 10 mL saline and storage at −20 °C. Final spore numbers were estimated by preparing serial dilutions of the spore suspensions in saline (0.85% NaCl) and plating 0.1 mL aliquots on Columbia blood agar (CBA) supplemented with 5% defibrinated horse blood and incubating anaerobically for 3 weeks at 4 °C.

#### 2.4.3. Preparation of Meat Samples, *C. estertheticum* Inoculation and Packing

Exactly 90 (10 × 10 × 1 cm) samples were prepared from *Biceps femoris* muscles (Charolais Cross heifers), purchased from a commercial beef processing plant. In a laminar flow unit, samples were spread inoculated with the prepared inocula to a final mean concentration of 10^3^ cfu·cm^−2^ and allowed to dry for 30 min at room temperature. The samples were then placed in individual bags with antimicrobial treatment or control bags containing a hydrogen sulphide strip (Sigma Aldrich, Gillingham, UK) and vacuum packed using the Vac Star S220 (Vac Star Shop, Sugiez, Switzerland). All samples were heat shrunk at 90 °C for 3 s and stored at 2 °C for 100 days in cardboard boxes in a chilling unit located in the on-site abattoir in Teagasc Food Research Centre (Dublin) The chiller temperature was monitored using an Easylog USB data logger (Lascar Electronics Ltd, Salisbury, UK) and the surface temperature of the samples was monitored using a Testo T-175 data logger.

### 2.5. Monitoring Vacuum Packs

Packs were visually examined every four days for the presence of gas and scored against the following criteria as described by Boerema et al. [15]; 0 (no gas bubbles in drip), 1 (gas bubbles in drip), 2 (loss of vacuum, considered to be the start of blown pack spoilage), 3 (“blown”), 4 (presence of sufficient gas inside the packs to produce pack distension) and 5 (tightly stretched, overblown packs or packs that are leaking).

### 2.6. Statistical Analysis

To obtain sufficient data for statistical analysis, five replicate samples were used for each antimicrobial treatment and five samples were used as treatment controls. The experiment was repeated on three separate occasions. Data on the time to the onset of blown pack spoilage, defined as the first day when each pack was assigned the score of 2, was analysed using GenStat Release 14.1 (VSN International Ltd, Hemel Hempstead, UK). Since all individual and pooled data failed the normality tests, data was analysed using the Mann—Whitney U (Wilcoxon rank-sum) test.

## 3. Results

**Figure 1:**
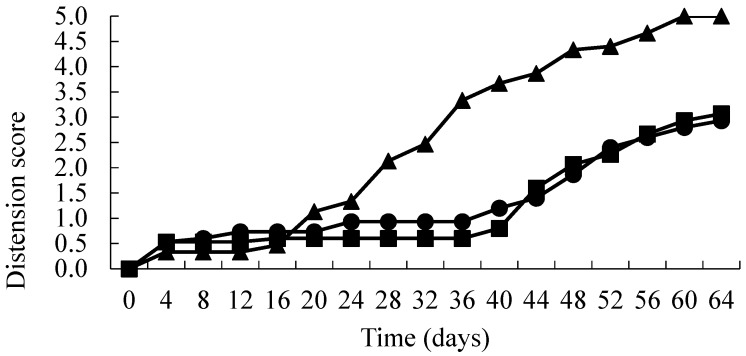
Distension status over time (days) of vacuum packs inoculated with spores of *C. estertheticum* and packaged in films containing 0 × MIC (▲), 2.5 × MIC (●) and 3.5 × MIC (■) AFV.

**Figure 2:**
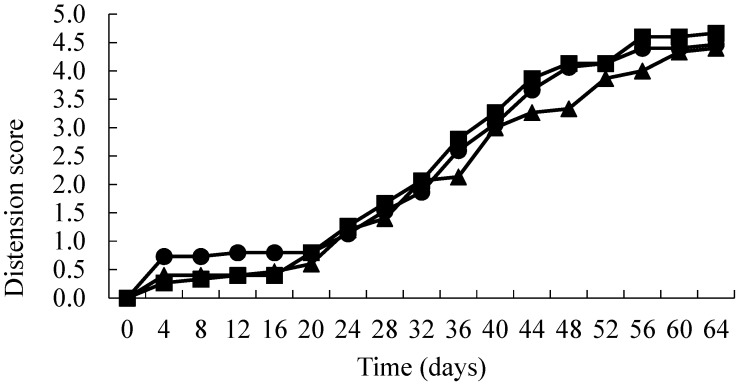
Distension status over time (days) of vacuum packs inoculated with spores of *C. estertheticum* and packaged in films containing 0 × MIC (▲), 2.5 × MIC (●) and 3.5 × MIC (■) IMDA.

**Figure 3:**
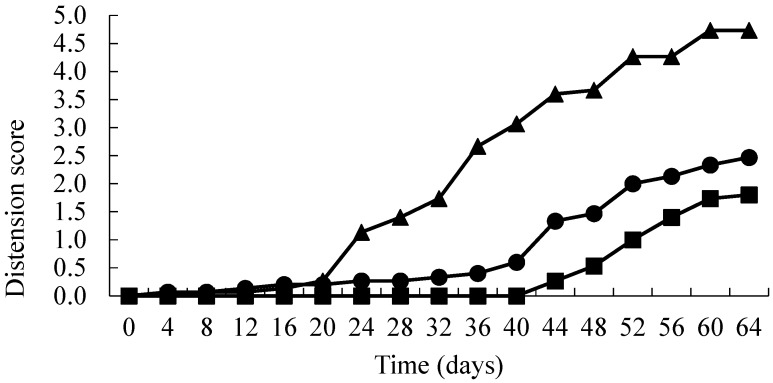
Distension status over time (days) of vacuum packs inoculated with spores of *C. estertheticum* and packaged in films containing 0 × MIC (▲), 2.5 × MIC (●) and 3.5 × MIC (■) SO.

The results are presented in Figure 1, Figure 2 and Figure 3. AFV active packs took significantly longer (*p* < 0.01) to spoil than the corresponding controls (Figure 1). This was primarily due to the onset of blown pack spoilage (score = 2) being delayed from approximately 28 days (control packs) to 48 days in the treated packs. Interestingly, there was no significant difference (*p* > 0.01) between the different concentrations of AFV used (2.5 and 3.5 times the MIC). In contrast, there was no significant difference in the IMDA treated films when compared to the control (Figure 2). Moreover, the time to the onset of blown pack spoilage was similar to that observed in the AFV control packs. The inoculated samples in SO treated packs showed a similar pattern to the AFV packs, as the time to spoilage in product wrapped in the treated films was significantly longer (*p* < 0.01) than the corresponding controls (Figure 3) and there was no significant difference (*p* > 0.01) between the different concentrations of SO used (2.5 and 3.5 times the MIC). Moreover, SO packs had significantly (*p* < 0.01) delayed blown pack spoilage as compared to AFV.

## 4. Discussion

Blown pack spoilage (BPS) is a global issue for the beef sector [16,17,18], including in Ireland where 0.8% of beef primals are contaminated with *C. estertheticum* [1]. Although meat spoiled in this way has no commercial value, control is reliant on sanitation of beef plants and equipment with a sporicidal agent such as peroxyacetic acid which is highly corrosive and often ineffective. Active packaging is a potential solution if suitable antimicrobials can be found.

Antimicrobial packaging incorporates an antimicrobial agent into a polymer film that prevents the growth of target microorganisms by extending the lag period, decreasing the live counts of microorganisms and/or reduces growth rate [19]. The antimicrobials used include organic acids, enzymes, bacteriocins, fungicides, polymers, natural extracts and essential oils [20]. However, the packaging methods and/or materials used are also important. Nisin, for example, incorporated into low-density polyethylene (LDPE) will suppress the growth of *Staphylococcus aureus* and *Listeria monocytogenes* [21], *Lactobacillus plantarum* when incorporated into soy protein and corn zein based films and *Salmonella Typhimurium* when coated onto polymeric films like PVC and nylon [22].

In this study, AFV and SO incorporated into active packaging films inhibited the growth of *C. estertheticum*, significantly retarding blown pack spoilage of beef primals. AFV contains bioflavanoids, citric, malic, lactic and caprylic acid, all of which have previously demonstrated antibacterial activity against Gram-positive bacteria [12,23,24]. Moreover, bioflavanoids are known to have antimicrobial activity against Clostridium spp. [25], possibly from the inhibition of membrane bound or intracellular proteins [26]. Although the exact antibacterial mechanisms of organic acids is not fully understood, it is assumed the undissociated form penetrates the cell, dissociates into anions and protons resulting in a decrease in cytoplasmic pH which inhibits a range of cellular functions [27]. Caprylic acid may also lower the pH of the cytoplasm disrupting the normal activity of intracellular enzymes [28] and has been shown to have antimicrobial activity against a range of foodborne bacterial pathogens including *Escherichia coli* O157, *Enterobacter sakazakii* and *L. monocytogenes* [29,30]. Interestingly, sodium octanoate (C_8_H_15_NaO_2_), is a derivative of caprylic acid (C_8_H_16_O_2_) and has similar antimicrobial properties [29,30].

In contrast, IMDA did not demonstrate anti-*estertheticum* properties when incorporated into the packaging film. This was unexpected as IMDA is composed of sodium diacetate, malic acid, mono and diglycerides of fatty acids, salt and excipients, all of which have previously been demonstrated to have anti-bacterial, including anti-Clostridium properties [27,31]. However, the effectiveness, or otherwise, of an anti-microbial compound incorporated into an active packaging film is dependent on a range of factors including the properties of the film/matrix and the characteristics of the food (pH, moisture, temperature, etc.). Thus, the apparent ineffectiveness of IMDA may be attributed to differences in important parameters such as release rate and reaction with the matrix (gelatine) [32].

## 5. Conclusions

In conclusion, the results of this study suggest that Auranta FV (AFV) and sodium octanoate (SO), incorporated in a gelatine matrix at concentrations of 2.5% or 3.5 times their MIC against *C. estertheticum* could be used in an active packaging system to prevent blown pack spoilage of beef primals.

## References

[1] Bolton D.J., Carroll J., Walsh D. (2015). A four-year survey of blown pack spoilage *Clostridium estertheticum* and *Clostridium gasigenes* on beef primal cuts. Lett. Appl. Microbiol..

[2] Broda D.M., Saul D.J., Lawson P.A., Bell R.G., Musgrave D.R. (2000). *Clostridium gasigenes* sp. nov., a psychrophile causing spoilage of vacuum-packed meat. Int. J. Syst. Evol. Microbiol..

[3] Adam K.H., Flint S.H., Brightwell G. (2010). Psychrophilic and psychrotrophic clostridia: Sporulation and germination processes and their role in the spoilage of chilled, vacuum-packaged beef, lamb and venison. Int. J. Food Sci. Technol..

[4] Cavill L., Renteria-Monterrubio A.L., Helps C.R., Corry J.E.L. (2011). Detection of cold-tolerant clostridia other than Clostridium estertheticum in raw vacuum-packed chill-stored meat. Food Microbiol..

[5] Dainty R.H., Edwards R.A., Hibbard C.M. (1989). Spoilage of vacuum-packed beef by a *clostridium sp*.. J. Sci. Food Agric..

[6] Moschonas G., Bolton D.J., Sheridan J.J., McDowell D.A. (2010). The effect of storage temperature and inoculum level on the time of onset of “blown pack” spoilage. J. Appl. Microbiol..

[7] Yang X., Gill C.O., Balamurugan S. (2010). Enumeration of *Clostridium estertheticum* spores in samples from meat plant conveyors and silage stacks by conventional and real time PCR procedures. Int. J. Food Saf..

[8] Moschonas G., Bolton D.J., Sheridan J.J., McDowell D.A. (2011). The effect of heat shrink treatment and storage temperature on the time of onset of “blown pack” spoilage. Meat Sci..

[9] Siragusa G., Cutter C., Willett J. (1999). Incorporation of bacteriocin in plastic retains activity and inhibits surface growth of bacteria on meat. Food Microbiol..

[10] Seydim A., Sarikus G. (2006). Antimicrobial activity of whey protein based edible films incorporated with oregano, rosemary and garlic essential oils. Food Res. Int..

[11] Cooksey K. (2001). Antimicrobial food packaging materials. Addit. Polym..

[12] Cruz-Romero M., Murphy T., Morris M., Cummins E., Kerry J. (2013). Antimicrobial activity of chitosan, organic acids and nano-sized solubilisates for potential use in smart antimicrobially-active packaging for potential food applications. Food Control.

[13] Clarke D., Molinaro S., Tyuftin A., Bolton D., Fanning S., Kerry J.P. (2016). Incorporation of commercially-derived antimicrobials into gelatin-based films and assessment of their antimicrobial activity and impact on physical film properties. Food Control.

[14] Lund B.M., Graham A.F., George S.M., Brown D. (1990). The combined effect of incubation temperature, pH and sorbic acid on the probability of growth of nonproteolytic type B *Clostridium botulinum*. J. Appl. Bacteriol..

[15] Boerema J.A., Broda D.M., Penney N., Brightwell G. (2007). Influence of peroxyacetic acid-based carcass rinse on the onset of “blown pack” spoilage in artificially inoculated vacuum-packed chilled beef. J. Food Prot..

[16] Lawson P., Dainty R.H., Kristiansen N., Berg J., Collins M.D. (1994). Characterization of a psychrotrophic Clostridium causing spoilage in vacuum-packed cooked pork: Description of *Clostridium algidicarnis* sp. nov.. Lett. Appl. Microbiol..

[17] Yang X., Gill C.O., Balamurugan S. (2009). Effects of temperature and pH on the growth of bacteria isolated from blown packs of vacuum-packaged beef. J. Food Prot..

[18] Silva A.R., Paulo E.N., Sant’ana A.S., Chaves R.D., Massaguer P.R. (2011). Involvement of *Clostridium gasigenes* and *Clostridium algidicarnis* in ‘blown pack’ spoilage of Brazilian vacuum-packed beef. Int. J. Food Microbiol..

[19] Han J.H. (2000). Antimicrobial food packaging. Food Technol..

[20] Malhotra B., Keshwani A., Harsha K. (2015). Antimicrobial food packaging: Potential and pitfalls. Front. Microbiol..

[21] Cooksey K. (2000). Utilization of antimicrobial packaging films for the inhibition of selected microorganisms. Food Packaging: Testing Methods and Applications.

[22] Natrajan N., Sheldon B.W. (2000). Efficacy of Nisin-Coated polymer films to inactivate Salmonella Typhimurium on fresh broiler skin. J. Food Prot..

[23] Burt S. (2004). Essential oils: Their antibacterial properties and potential applications in foods—A review. Int. J. Food Microbiol..

[24] Batovska D., Parushev S., Stamboliyska B., Tsvetkova I., Ninova M., Najdenski H. (2009). Examination of growth inhibitory properties of synthetic chalcones for which antibacterial activity was predicted. Eur. J. Med. Chem..

[25] Wu X., Alam Z., Feng L., Tsutsumi L.S., Sun D., Hurdle J.G. (2014). Prospects for flavonoid and related phytochemicals as nature-inspired treatments for Clostridium difficile infection. J. Appl. Microbiol..

[26] Cushnie T.P., Lamb A.J. (2011). Recent advances in undersatdning the antibacterial properties of flavonoids. Int. J. Antimicrobiol. Agents.

[27] Ricke S.C. (2002). Perspectives on the use of organic acids and short chain fatty acids as antimicrobials. Poult. Sci..

[28] Sun C.Q., O’ Conner C.J., Robertson C.J. (2002). The antimicrobial properties of milk fat after partial hydrolysis of calf pregastric lipase. Chem. Biol. Interact..

[29] Annamalai T., Nair M.K.M., Marek P., Vasudevan P., Schreiber D., Knight R., Hoagland T., Venkitanarayanan K. (2004). In vitro inactivation of enterohemorrhagic *Escherichia coli* O157:H7 in bovine rumen fluid by caprylic acid. J. Food Prot..

[30] Nair M.K.M., Vasudevan P., Hoagland T., Venkitanarayanan K. (2004). Inactivation of *Escherichia coli* O157:H7 and *Listeria monocytogenes* in milk by caprylic acid and monocaprylin. Food Microbiol..

[31] Juneja V.K., Thippareddi H. (2004). Inhibitory effects of organic acid salts on growth of *Clostridium perfringens* from spore inocula during chilling of marinated ground turkey breast. Int. J. Food Microbiol..

[32] Appendini P., Hotchkiss J.H. (2002). Review of antimicrobial food packaging. Innov. Food Sci. Emerg. Technol..

